# Enhancing the Efficiency of a Radiation Oncology Department Using Electronic Medical Records: Protocol for Preparing Radiotherapy

**DOI:** 10.2196/51002

**Published:** 2024-02-23

**Authors:** Hao-Shen Cheng, Weir-Chiang You, Ni-Wei Chen, Mu-Chih Hsieh, Che-Fu Tsai, Chia-Jing Ho, Chien-Chih Chen

**Affiliations:** 1 Taichung Veterans General Hospital Taichung Taiwan

**Keywords:** efficiency, electronic medical records, Hospital Information System, protocol, radiation oncology

## Abstract

**Background:**

Electronic medical records (EMRs) streamline medical processes, improve quality control, and facilitate data sharing among hospital departments. They also reduce maintenance costs and storage space needed for paper records, while saving time and providing structured data for future research.

**Objective:**

This study aimed to investigate whether the integration of the radiation oncology information system and the hospital information system enhances the efficiency of the department of radiation oncology.

**Methods:**

We held multidisciplinary discussions among physicians, physicists, medical radiation technologists, nurses, and engineers. We integrated paper records from the radiation oncology department into the existing hospital information system within the hospital. A new electronic interface was designed. A comparison was made between the time taken to retrieve information from either the paper records or the EMRs for radiation preparation. A total of 30 cases were randomly allocated in both the old paper-based system and the new EMR system. The time spent was calculated manually at every step during the process, and we performed an independent 1-tailed *t* test to evaluate the difference between the 2 systems.

**Results:**

Since the system was launched in August 2020, more than 1000 medical records have been entered into the system, and this figure continues to increase. The total time needed for the radiation preparation process was reduced from 286.8 minutes to 154.3 minutes (*P*<.001)—a reduction of 46.2%. There was no longer any need to arrange for a nurse to organize the radiotherapy paper records, saving a workload of 16 hours per month.

**Conclusions:**

The implementation of the integrated EMR system has resulted in a significant reduction in the number of steps involved in radiotherapy preparation, as well as a decrease in the amount of time required for the process. The new EMR system has provided numerous benefits for the department, including a decrease in workload, a simplified workflow, and conserving more patient data within a confined space.

## Introduction

The advantages of electronic medical records (EMRs) include facilitating medical process management, quality control, and the sharing of medical record data among different departments in a hospital, as well as reducing both costs and storage space for paper medical records [1,2]. The Ministry of Health and Welfare in Taiwan implemented a pilot program for the digitalization of medical records in 2002, and our hospital participated in this program to digitalize our medical records.

However, due to the highly complex nature of the information contained in radiotherapy (RT) treatment plans [3,4], the paper records of RT are not easily integrated into the hospital information system (HIS) [5-7], causing operational difficulties for medical staff across different departments. These difficulties included obtaining information such as a patient’s cumulative dose, fractionations, and frequency of treatment through our HIS. Additionally, searching for treatment history in paper records is a time-consuming and inefficient process that can lead to errors, especially as the records are handwritten. Moreover, the software used in a linear accelerator is independent of the HIS in most radiation oncology departments. Doctors and other medical personnel must use 2 different systems, which means they often have repeated tasks. For example, when a physician wants to write an RT certificate for a patient, information such as date of treatment, total dose, and frequency of treatment must be entered into a certificate manually, which is prone to errors and is time-consuming. Integrating the 2 systems would allow doctors to work more efficiently and serve more patients. Structured data can also be used for future quality control and clinical medical research. Furthermore, various monthly and annual reports in an integrated system can allow the department manager to effectively monitor the clinical load in the department, and the data can be used as a reference for manpower deployment and overtime budget application [8].

In line with national policy and the needs of our hospital, an EMR system for the radiation oncology department was established. This study examines how the integration of the RT EMR system with our HIS improved the efficiency of our department and the accuracy of the data. In this study, we demonstrated that by consolidating the 2 separate systems into 1, we were able to significantly increase efficiency, save time, and conserve valuable resources.

## Methods

### Overview

In January 2020, the first meeting was convened to discuss the implementation of EMRs. Representatives from various staff groups, including physicians, physicists, medical radiation technologists, nurses, and engineers, were appointed to discuss workflow arrangements and functional requirements. The preliminary system was completed in July 2020, followed by internet-based testing and debugging. The updated system was fully deployed in August 2020, replacing the old system entirely. We kept a monthly review of the system in order to debug or improve the user experience.

The first step involved reviewing the paper documents, including graphs, tables, and written records. Staff from different departments held monthly discussions in the Radiation Oncology Department conference room, and the records of our meetings were saved in our database for the future. Our staff and engineers discussed the feasibility of transforming each item into electronic records. In the paper documents, check marks were made under the date in the table upon completion of daily treatment. If daily treatment was not performed, the field would be left blank. The total number of treatment days was calculated manually. Following discussions with the engineers, key information, such as the radiation beam energy, radiation technique, planned target volume and dose, treatment date, and daily treatment status, were selected as items to be included in the electronic records.

The subsequent step involved educating physicians about how to input treatment plan information into the HIS. A new electronic interface was designed for physicians to input crucial data, such as the treatment intent, planned target volume, planned dose, and planned number of treatment days. These data would then be integrated into the EMR.

**Figure 1:**
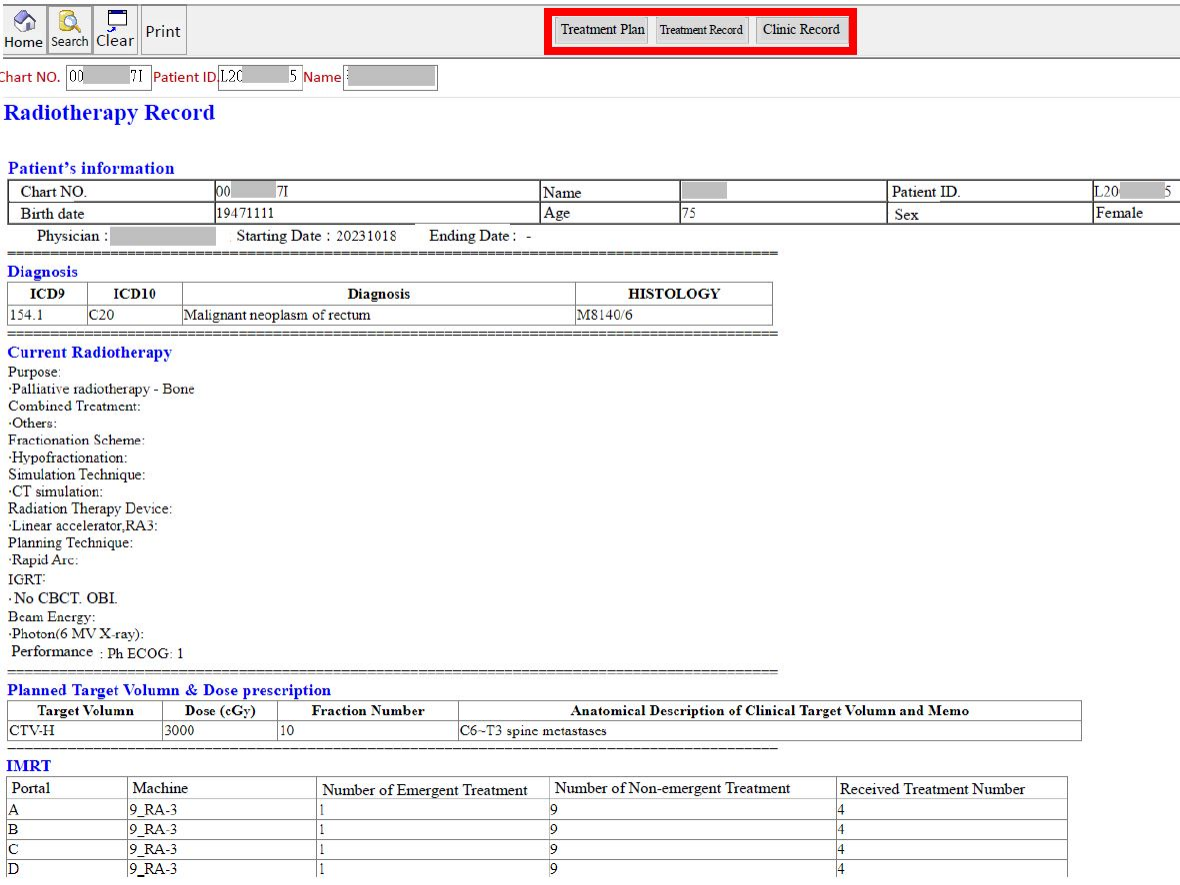
The final version of the electronic medical record (EMR), including the records of the past treatment of patients, a review of their past treatment status, and a link to the latest EMRs for tracking their current condition. CBCT: cone-beam computed tomography; CT: computed tomography; ICD9: International Classification of Diseases, Ninth Revision; ICD10: International Statistical Classification of Diseases, Tenth Revision; OBI: on-board imaging.

In addition, daily treatment progress was recorded automatically in the HIS. The total number of treatment days was controlled by the electronic treatment plan entered by the physician, thus preventing unnecessary dose delivery. The EMR retrieved the actual treatment dates from the HIS, thereby providing precise treatment progress information. After several adjustments, the final version of the EMR system was launched in the HIS (Figure 1).

**Figure 2:**
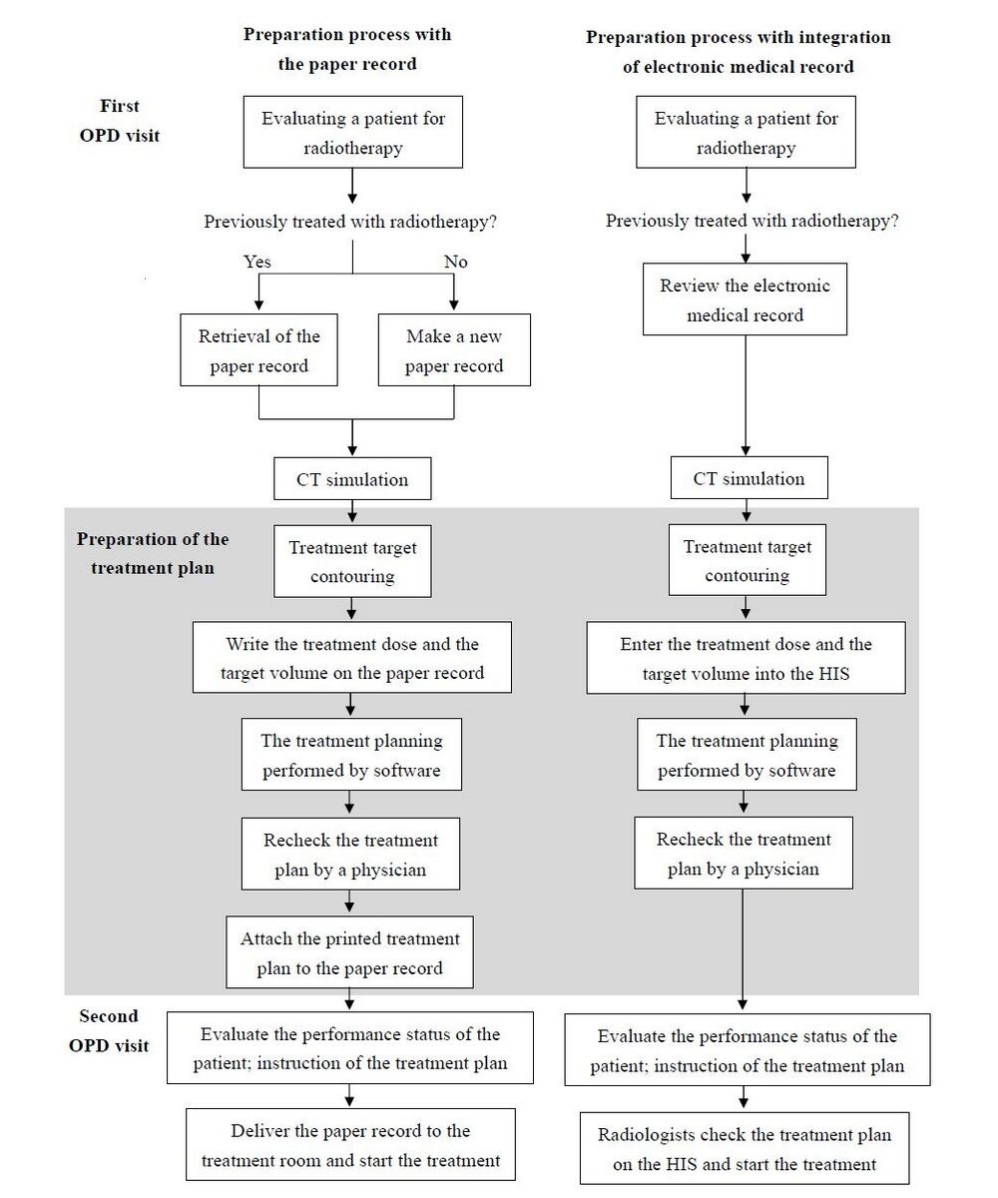
Comparison of the preparation processes between paper record usage and the integrated electronic record system. The integration of the electronic record system has led to a significant reduction in the time required for the radiation treatment preparation process. CT: computed tomography; HIS: hospital information system; OPD: outpatient department.

Figure 2 shows the comparison of the preparation processes between paper record usage and the integrated electronic record system. In order to evaluate the results of our integration, a comparison was made between the time taken to retrieve information from either the paper records or the EMRs for radiation preparation. A total of 30 cases were randomly allocated in both the old paper-based (legacy) system and the new EMR system. The time spent was calculated manually at every step during the process, and we performed an independent *t* test to evaluate the difference. All statistical analyses were performed with SPSS (version 23; IBM Corp). A *P* value of <.05 was considered statistically significant. This study explores the benefits of converting paper medical records in the radiation oncology department into EMRs and integrating them with the hospital’s existing electronic medical systems. It does not involve personal information or related ethical issues.

### Ethical Considerations

Our research mainly focuses on the integration of the EMR system into the HIS and the efficiency of our new protocol in preparing RT records for new patients. The study does not involve personal or sensitive information related to human participants, so privacy and confidentiality protection considerations for human participants are not applicable [9]. There is no image or identifiable information related to an individual patient.

## Results

**Figure 3:**
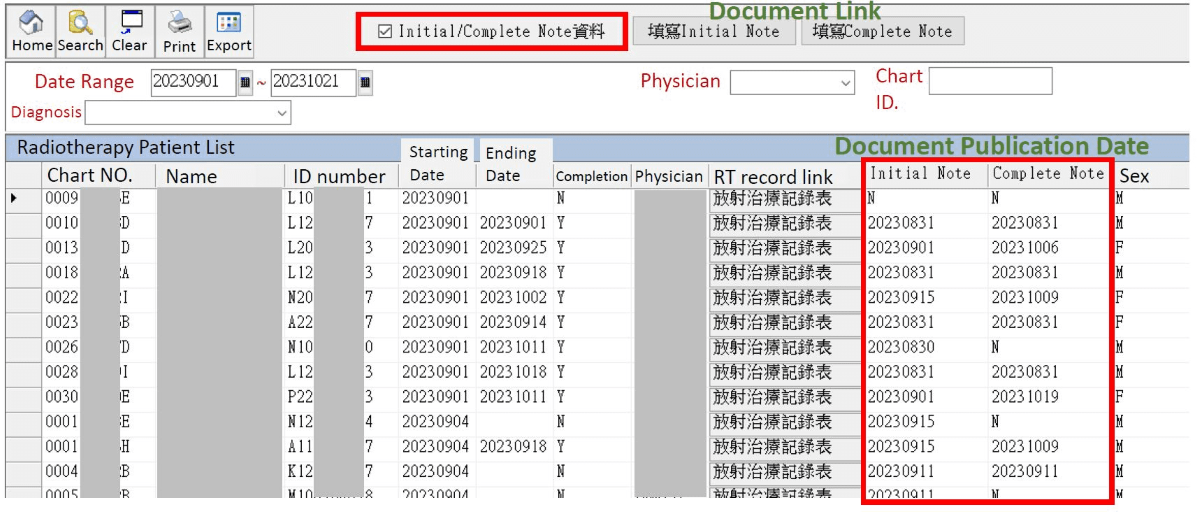
The list of patients and their related information created by our electronic medical record. Integration of the system allowed the patient lists and their related information to be displayed, allowing physicians to easily check the treatment status of each patient. RT: radiotherapy.

Since launching in August 2020, the system has been highly used, with the number of medical records entered exceeding 1000 and continuing to increase. Integration of the system has meant that patient lists and their related information are now displayed (Figure 3), allowing physicians to easily check the treatment status of each patient. Physicians can also query the records of past treatments of patients, review their past treatment status, and link their latest EMRs to track their current condition. The new system can also export the files of all patients who have been treated in the past for the purpose of statistical analysis. Additionally, the number of treatment days for each patient can now be automatically calculated. The medical record writing format is now consistent among physicians.

As shown in Figure 2 the integration of the electronic record system has led to a significant reduction in the time required for the radiation treatment preparation process. The new protocol post integration of the EMR streamlines procedures by obviating the need for retrieving previous paper records or generating new ones. Physicians input treatment information directly into the HIS instead of transcribing it onto paper records, eliminating the necessity for printing treatment plans. In addition, physicians can scrutinize the treatment plan and monitor treatment progression seamlessly through the HIS. Radiologists, in turn, can access the treatment plan on the HIS and commence patient treatment, thereby economizing time previously spent awaiting the transportation of paper records. Specifically, the processing time has decreased from 286.8 minutes to 154.3 minutes (*P*<.001), representing a total reduction of 46.2%.

## Discussion

### Overview

Before the implementation of the EMR system, retrieving RT history was conducted manually, which was time-consuming and burdened nurses with a significant workload. The integration of the electronic system has simplified the process by eliminating several procedures and allowing physicians to instantly access RT records. Many new functions have also been added to this system.

First, the establishment of the new scheduling system allows physicians to modify the treatment schedules of new patients at any time. All staff in the department have the authority to modify the scheduling system. Second, the scheduling system has a hyperlink on the interface, which allows a doctor to view the patient’s schedule while browsing the medical records, as well as edit the patient’s RT notes. Third, a doctor can easily check the patient’s status on the system. The patient list generated by the system can show information about a patient, including contact information, RT starting date or ending date, the number of treatments, and the completion status of RT notes.

The new electronic system has brought several benefits. First, it facilitates communication between physicians and other staff within the department. Clinical RT requires both intensive and extensive communication and cooperation among various groups of staff, including physicians, physicists, medical radiation technologists, nurses, and administrative personnel, in order to proceed smoothly. It is crucial that communication be both correct and instantaneous. With our system, a physician can enter the handover of a patient into the system, and a radiologist can read the handover before the treatment. Staff from different departments can also find the treatment details of a patient on the HIS.

Second, there is no longer a need to arrange for a nurse to organize the RT paper records, which saves a workload of 16 hours each month. Aguirre et al [2] concluded that electronic health records can minimize delays, increase health care workers’ satisfaction, and decrease the chances of usability being compromised. Our study also showed that the integration of the radiation oncology EMRs and the HIS simplifies the workflow at our clinic (Figure 2), saving time spent performing radiation preparation procedures and enhancing efficiency. The protocol covered seamless transitions from one stage of the process to the next, ensuring that the entire workflow remained in compliance with standards for RT preparation. Furthermore, Chen et al [10] and Huang et al [11] showed that shortening the preparation time needed before the start of RT improved the treatment effect. Previous research has also shown that, with the assistance of an information system, the work process becomes smoother. Physicians can be given an automatic reminder or warning, which, in turn can shorten the necessary work preparation time from 12.2 days to 8.9 days [12].

Third, the statistical functions of this information system allowed the manager of the department to obtain accurate information on the number of people treated by each machine, as well as the number of fields per day, the number of simulations, the number of brachytherapy procedures, the number of people waiting for treatment, and the waiting time for each patient. The workload for each physician can also be monitored, and various monthly and annual reports allow the department manager to effectively trace the clinical workload in each treatment room. Monthly and annual reports can also be used as a reference baseline for manpower deployment and overtime budget applications [8]. Having mastery over these data can contribute to a better understanding of the operation of the department, and the information provided by the system can serve as important reference data for decision-making on issues such as whether to purchase new machines or modify manpower.

Additionally, the new system can assist with medical summary writing and education for a resident doctor. Each RT summary requires an average processing time of 10-15 minutes. Currently, with the new system, it only takes about 5 minutes to complete an RT summary because the detailed information on RT is automatically retrieved from the EMR. Doctors simply need to fill in the information about side effects and the treatment response. The system can also remind physicians to finish the initial note and to complete the RT summary when treatment is completed. For educational purposes, an electronic RT summary allows a resident doctor to quickly review the modifications from an attending physician and become familiar with the key points of the appropriate medical record [6,13].

Finally, at least 39,600 sheets of paper were used to prepare RT paper records per year, which is equal to 713 kg of carbon emissions. Furthermore, the demand for storage space tends to increase annually for paper records, but electronic records only require a relatively small physical space to store a large amount of patient data. The implementation of the improved EMR system has freed up space in our department that can now be put to better use. On the other hand, the number of our new patients increased annually. In 2020, we accommodated 1920 new patients, attended to 2118 newcomers in 2021, and extended our services to a total of 2230 fresh patients in 2022. Because the EMR system has decreased the number of radiation therapy transactions per day, physicians and nurses can manage clinical matters more efficiently, allocating more time for meaningful communication with patients. Furthermore, our department is relieved of the necessity to expand storage space for paper records as the EMR system adeptly conserves extensive patient data within a more confined space.

The system offers great flexibility for future expansion and can adapt to changes in clinical practice. We plan to improve the system by introducing an automatic patient registration system, a face recognition system, and an artificial intelligence scheduling system.

### Limitations

The challenge of transitioning from paper records to electronic records remains high. The process is time-consuming and requires multidisciplinary team discussions. One limitation of our study is that the multidisciplinary team discussions only included engineers and staff in our department. Ideally, feedback and opinions should also have been collected from other departments in our hospital to improve our EMR system and make it more user-friendly. A survey to assess satisfaction with the new system will be conducted in the future. Any future studies should also have a larger sample size to validate our results. Furthermore, a comparison with other radiation oncology departments would be insightful.

### Conclusion

The implementation of the integrated EMR system has resulted in a significant reduction in the number of steps involved in RT preparation, as well as a decrease in the amount of time required for the process. The new EMR system has provided numerous benefits for the department, including a decrease in workload, a simplified workflow, and conserving more patient data within a confined space.

## Abbreviations

EMR: electronic medical record
HIS: hospital information system
RT: radiotherapy

## Data Availability

The data sets generated and analyzed during this study are available from the corresponding author on reasonable request.
